# Dietary Pattern and Macronutrients Profile on the Variation of Inflammatory Biomarkers: Scientific Update

**DOI:** 10.1155/2018/4762575

**Published:** 2018-03-14

**Authors:** Brenda Kelly Souza Silveira, Thatianne Moreira Silva Oliveira, Patrícia Amaro Andrade, Helen Hermana Miranda Hermsdorff, Carla de Oliveira Barbosa Rosa, Sylvia do Carmo Castro Franceschini

**Affiliations:** Department of Nutrition and Health, Universidade Federal de Viçosa, Viçosa, MG, Brazil

## Abstract

It is known that the dietary pattern and macronutrients profile may influence the expression and secretion of inflammatory biomarkers, and the low-grade inflammation is associated with the manifestation of noncommunicable chronic diseases. Therefore, this review aimed to present and discuss the role of dietary patterns and macronutrients on the variation of inflammatory markers related to NCD risk. Scientific evidences within the last five years based on clinical trials, case-controls, cohorts, and cross-sectional studies indicate that normocaloric, carbohydrate-moderated, low-glycemic index, protein-moderated, monounsaturated and polyunsaturated fatty acid-rich, omega-3, and low-saturated fat diets display positive effects on the inflammatory state, both in healthy individuals and in those with cardiovascular risk, although the second group seems to benefit more from changes in the dietary profile.

## 1. Introduction

Low-grade inflammation refers to a series of metabolic and physiological modifications with proinflammatory cytokines and increased oxidative stress [1]. It is related to the development and grievance of noncommunicable chronic diseases (NCD), and one of the factors associated with the low-grade inflammation is the intake of foods with proinflammatory characteristics [2, 3].

The dietary pattern may be defined as a combination of foods frequently consumed by individuals and population groups [4]. The importance of dietary pattern analysis lies in the fact that the observed effect for a given nutrient or food in environmentally controlled investigations differs from the effect found within the context of different dietary patterns routinely adopted by populations [5].

Indeed, some dietary patterns, such as the Mediterranean and DASH (Dietary Approach to Stop Hypertension) diets, are associated with an improved weight control and lower incidence of NCD [6, 7] so that they have been employed as reference or “healthy” diets by the scientific literature and clinical-nutritional practice. Furthermore, the dietary pattern has been investigated as a potential modulator of inflammatory markers [8]. Thus, the adoption of healthy dietary patterns distinguished by high intake of fruits, vegetables, and low intake of sugar and fats (saturated and trans) seems to decrease the risk of NCD through its influence on the related low-grade inflammation [7, 9].

On the other hand, the impact of the distribution of macronutrients—carbohydrates, proteins, and lipids—on the inflammatory state has been extensively investigated [10–15], whilst the amount and origin of the nutrients ingested have played an important role in the development of the low-grade inflammation [16]. Therefore, this review aimed to present and discuss the role of dietary patterns and macronutrients on the variation of inflammatory markers related to NCD risk.

## 2. Materials and Methods

For this review, the electronic databases PubMed (National Library of Medicine, Bethesda, MD) and ScienceDirect were researched. The following keywords were used for article search: “dietary fat”; “fatty acids,” “omega-3,” “saturated fat,” “olive oil,” “dietary patterns,” “dietary protein,” and “dietary carbohydrate” always combined with the MeSH terms “inflammation” or “inflammatory markers.” The search was limited to English studies published between March 2012 and April 2017. The inclusion criteria considered original studies published within the last 5 years, in English, which assessed the relation of dietary pattern and macronutrients profile on the variation of inflammatory biomarkers, in observational or interventional study designs. Review papers, editorials and book chapters, case reports, studies with animals or cells, studies that did not aim to evaluate the inflammation, studies that used supplements with registered mark, and studies that assessed the relation between inflammation and diseases other than diabetes, dyslipidemia, metabolic syndrome, obesity, and cardiovascular diseases were excluded from the present review.

Initially, 9429 papers were found. Of these, 9183 papers were excluded after title and abstract screening, and 246 papers were fully read. Of these, 40 papers met the inclusion criteria and were kept for this review (Table 1). In order to complement the discussion, other papers related to the issue addressed were used, despite the year of publication.

## 3. Results and Discussion

### 3.1. Dietary Patterns

The dietary patterns analysis helps to understand the factors that contribute to NCD [17, 18]. Therefore, in the last decades, several authors have been dedicated to investigate the association between dietary patterns and indices that evaluate the quality of the diet with inflammatory markers instead of investigating isolated nutrients [3, 19, 20]. The analysis of dietary pattern can be *a priori*, when a score is attributed to the diet according to previous knowledge concerning dietary disease health outcomes, or *a posteriori*, when statistical techniques are used to explore new patterns [21].

Among *a priori* techniques, several indexes have already been developed, such as the Mediterranean Diet Score [22], Healthy Eating Index [23], Diet Quality Index [24], and Dietary Inflammatory Index [25]. Briefly, these indexes assess the conformity of individuals' diet to guidelines and dietary recommendations by assigning an interpretable score and have been adapted to make improvements and meet the specificities of each population [26–28].

On the other hand, the cluster and factor analyses are the most frequently used *a posteriori* techniques [29, 30]. They allow, respectively, to group individuals with similar food intake or to establish standards from correlating foods. Both approaches, *a priori* and *a posteriori*, have limitations and positive aspects, so there is no better technique and they are used to investigate different questions [21].

Regardless of the approach used, it is a consensus that dietary patterns deemed healthy displays as an inverse relationship with the concentrations of inflammatory biomarkers [5, 31, 32]. Dietary patterns that potentially combat the inflammatory state and reduce the risk for NCD are characterized by the elevated intake of vegetables, green leaves, whole grain cereals, fruits, chestnuts, fish and olive oil, low consumption of embedded meat, sugary drinks, processed foods, and saturated fat [33, 34].

In this context, Corley et al. [13] defined two types of dietary patterns: the “Mediterranean diet pattern” and the “conscious consumption pattern,” which involves higher fruit consumption and low consumption of meat, eggs, and liqueurs. Inverse associations were observed between fibrinogen and Mediterranean diet. In turn, the individuals who obtained higher scores in the “conscious consumption pattern” displayed lower serum concentrations of C-reactive protein (CRP) (≤3 mg/L). In this study, both patterns were considered healthy, thus reaffirming the importance of diversifying foods for health maintenance. For the elderly, a higher consumption of fruits, vegetables, fowl, fish, and low-fat dairy products is related to lower systemic inflammation [30].

On the other hand, in the study by Bédard et al. [12] with an adult population, the introduction of a dietary pattern in the Mediterranean diet was monitored during a four-week period, with the purpose of evaluating potential modifications in the serum concentrations of CRP. Men who displayed elevated CRP concentrations (≥2 mg/L) showed a reduction in concentrations during the study, while an increase was observed in men who presented normal concentrations (<2 mg/L). In general terms, this study did not report effective results by the Mediterranean diet, which may be related to the reduced follow-up time, diet composition, and control of variables that might have influenced the participants' inflammatory state. Hermsdorff et al. [35] conducted an intervention with adult individuals during eight weeks, in which the adherence to the Mediterranean diet occasioned reduction in the CRP concentrations as well as other inflammatory markers. These results corroborate other interventional studies [36, 37], thus indicating the anti-inflammatory role of the Mediterranean pattern.

In turn, the occidental dietary pattern, characterized by the excessive intake of calories, salt, fat, and sugar, has been associated with the increase of low-grade inflammation and with the development of the NCD [38, 39]. Within the adult population, the following patterns were identified: “pastas,” “sandwich,” “starchy vegetables,” “sugary drinks,” “dessert,” “breads,” “fowl,” “frozen food,” “alcoholic beverage,” and “pizza.” After the analysis of the inflammatory markers, all patterns displayed elevated CRP (>3 mg/L) at the same time as the highest average was that of the “frozen food” pattern [40].

A similar effect was observed in the study by Kong et al. [5], who evaluated the food intake of overweight individuals, whereas three dietary patterns were identified, according to similarity groups: “high intake of sugar, fat, and salt” (Group 1), “higher intake of water, yogurt, cereals, eggs, and chestnuts” (Group 2), and “higher intake of fruits, yogurts, soups, and lower sugar intake” (Group 3). Differences regarding the inflammatory markers—CRP and interleukin 6 (IL-6)—were not observed between the groups. However, after the evaluation of the concentrations of CD136—a transmembrane protein found in the adipose tissue, with anti-inflammatory function—Group 3 displayed higher concentrations.

Similarly, when considering the foods altogether, we can stablish how anti- or proinflammatory a diet can be [8, 41]. In this context, Ozawa et al. [15] conducted a study with adults and gave factorial weights to the foods, thus indicating how pro- or anti-inflammatory they were. From this distribution, participants' diets were assessed, and those who reported dietary patterns considered inflammatory (higher consumption of red meat and processed and fried foods), consequently displayed higher IL-6 levels. The interconnection between nutrients and health should not be assessed in isolation, once it is known that nutrients from the foods act synergistically in the body and may interact with each other [42].

The foods included in a diet may directly influence the individual's health. Fruits and vegetables are foods that, when consumed on a daily basis in an adequate amount, are capable of reducing the oxidative stress and low-grade inflammation markers [9, 43].

Lee et al. [32], after studying an adult population, identified different dietary patterns, named “coffee pattern,” “fruit pattern,” “meat pattern,” and “vegetable pattern.” The individuals with a higher score for the “vegetable pattern” displayed a lower CRP concentration, as well as a higher antioxidant intake. A similar result was reported by McGeoghegan et al. [44], who identified two dietary patterns: “1” and “2”, these being the ones with higher and lower antioxidant and anti-inflammatory loads, respectively. Pattern “1” was inversely associated with the CRP concentrations and lower prevalence of diabetes.

It is known that fruits and vegetables have various bioactive and antioxidant compounds [45–47] and that, when associated with whole grains, fish, and lean meat, may reduce the risk factors with respect to the development of several diseases [48]. This association was verified in the study by Abete et al. [49], who investigated a population of healthy individuals with a history of stroke and identified the patterns named “healthy” and “not healthy.” Healthy individuals showed higher adherence to the “healthy” pattern and displayed lower CRP concentrations. In addition, the adherence to the “healthy” pattern was a protective factor for the development of stroke.

The studies presented in this review (Table 2) showed the importance of an integral nutrition, particularly to base health promotion actions. The adherence to patterns deemed healthy, such as the Mediterranean diet, and patterns that included fruits and vegetables and reduced intake of processed food has been associated with the reduction of inflammatory and systemic markers, as well as with the risk of NCD [50]. Therefore, it is possible to verify that a balanced and varied nutrition, rich in natural foods and with the least amount of processed foods, is capable of reducing the low-grade inflammation and associated diseases. In addition, it was possible to observe that the dietary patterns identified display similar characteristics and effects, regardless of the population, thus highlighting the importance of assessing the cumulative associations of certain groups of foods.

### 3.2. Lipids

The proportion and type of fats included in the diet influence the degree of inflammation and the risk of NCD. In this context, the intake of polyunsaturated fatty acids (PUFA), particularly the long-chain ones from the w-3 series (n-3 PUFA), is related to the reduction of the risk of cardiovascular diseases and death due to its anti-inflammatory potential [51–54]. Diets high in monounsaturated fatty acids (MUFA) have also been highlighted for their potential in reducing inflammation due to the lower expression of genes related to the synthesis of IL-6 [10, 55].

Saturated fatty acids (SFA), on the other hand, favor the increase of inflammation. Most studies included in this review related the consumption of SFA to the increase of inflammatory markers and NCD. Kantor et al. [56], in a retrospective cohort with elderly, observed that the lower ingestion of SFA was associated with the reduction of CRP in eutrophic and overweight groups, but not in obese, thus suggesting that the modulating role of the SFAs in the CRP concentrations is mediated by the individual's nutritional state. Lesná et al. [57] found reduction of CRP and IL-8 in a three-week randomized crossover study, after replacement of an SFA-rich diet (42% of the total caloric value “TCV”) by a PUFA-rich diet (40% of the TCV). The increase in the expression of proinflammatory cytokines associated with the consumption of SFA is due to the activation of toll-like receptors (TLR), in particular, the TLR-4. Therefore, transcription factors such as the factor nuclear kappa B (NF-*κβ*) are stimulated, resulting in the synthesis of proinflammatory cytokines [8, 58].

Silver et al. [59] investigated the effect of a hyperlipidemic diet (50% of the TCV), with equal proportions of MUFA, PUFA, and SFA, on overweight individuals, and observed, after two weeks, an increase of 6% in fat oxidation, an average reduction of 2.5 kg of body fat, an average increase of 2.5 kg of lean mass, and a reduction in the interleukins IL-1*α*, IL-1*β*, 1L-12, and IL-17, besides interferon gamma (IFN-*γ*), tumor necrosis factor-alpha (TNF-*α*), and tumor necrosis factor-beta (TNF-*β*) concentrations and improvement in vascular function. In this context, hypolipidemic diets may not be the best strategy to reduce NCD, as the fatty acids profile (MUFA, PUFA, and SFA) is more relevant for modulating the inflammation than the proportion of total lipids in the diet. In addition, diets with higher lipid content are more palatable and provide greater satiety and better weight control [60].

With respect to the linolenic acid, an intervention with type-2 diabetics resulted in reduction in the IL-2 concentrations and TNF-*α* after n-3 supplementation (eicosapentaenoic “EPA” 1.548 mg/d; docosahexaenoic “DHA” 828 mg/d; and others n-3 338 mg/d) during eight weeks, although CRP levels were maintained [61]. Rajkumar et al. [62] reported an improvement in the proinflammatory profile with CRP reduction in overweight patients after n-3 supplementation (EPA 180 mg/d and DHA 120 mg/d) during six weeks. Ito et al. [63] observed the same effect with dyslipidemic individuals. In turn, Itariu et al. [64] verified a reduction in the gene expression of inflammatory biomarkers in the subcutaneous adipose tissue, a reduction in IL-6 concentration, and increased anti-inflammatory eicosanoids in the visceral adipose tissue, after n-3 supplementation (EPA 460 mg/d and DHA 380 mg/d) during eight weeks. In another study, the authors observed a CRP reduction after eight weeks of n-3 supplementation (EPA 720 mg and DHA 480 mg) in men with coronary artery disease [65].

Accordingly, the beneficial mechanism of the linoleic acid (n-3 series) has been related to its derivatives, the EPA C20 : 5 n-3 and DHA 22 : 6 n-3 acids, which have a higher anti-inflammatory effect than the derivatives of the arachidonic acid (AA n-6 PUFA) derived from the linoleic acid (n-6 series). Such a effect was reported in a study with adults, in which an increase was reported in the formation of EPA and DHA after four months of supplementation (1.4 g/d) in comparison with the placebo group (soy oil), although with no changes in AA metabolites [66]. Hence, the cause of reduction in inflammation was the synthesis of anti-inflammatory metabolites, rather than the suppression of proinflammatory metabolites derived from the AA.

However, investigations about the effect of n-3 lipids on NCD-related biomarkers reported contradictory findings. The cardiometabolic risk of the sample population seems to influence the results, as the beneficial effects are more evident in individuals with more than one risk factor, rather than excessive body fat alone [67, 68]. Studies in which the population had metabolic syndrome (MS), diabetes mellitus (DM), and/or dyslipidemia [32, 61, 63] reported more pronounced results in the improvement of proinflammatory biomarkers than studies with individuals who were overweight only, with no other associated diseases/risk factors [67–69]. Indeed, some studies did not find evidences that justify the n-3 supplementation [67, 68, 70–72]. Dewell et al. [70] assessed the effect of a low- (2.2 g/d) and high-dose (6.6 g/d) plant-derived n-3 supplementation, compared to the low- (1.2 g/d) and high-dose (3.6 g/d) sea-derived n-3 supplementation but did not find differences between the groups for IL-6 and soluble intercellular adhesion molecule-1 (sICAM-1), after eight weeks. Nigam et al. [71] assessed the effect of n-3 supplementation (4 g/d) during 271 ± 129 days on average and reported that, after 6 months, the reduction of CRP and myeloperoxidase (MPO) was similar to that observed in the placebo group. Darghosian et al. [72] also observed the maintenance of IL-6, IL-8, IL-10, TNF-*α*, and monocyte chemoattractant protein after 6 months of n-3 supplementation (4 g/d). Different proportions of n-3 in the diet did not affect the IL-6, monocyte chemoattractant protein-1 (MCP-1), concentrations TNF-*α* 1 and 2 receptors, and PCR, after 14 weeks of n-3-rich diet (3.5% of TCV), compared to the group with a diet poor in n-3 (0.5% of TCV) [67].

The differences observed in the inflammatory profile may be related to variations in the intervention time and in the supplemented daily dose. The daily doses used varied between 1 and 6.6 g/d, whereas doses above 3 g/d were less common. Another aspect worth considering is the origin of fatty acids of the n-3 series, which may be plant or sea-derived. Only one study included in this review, conducted by Dewell et al. [70] assessed the effect of different n-3 sources, although a beneficial effect was not found in any of the inflammatory markers evaluated, regardless of the dose or source. However, since most of the studies did not find evidences of health damage caused by n-3 supplementation and most of them reported benefits from its use, the usual intake of foods that are source of n-3 may be a strategy to reduce risk or grievance of diseases associated with the inflammation, particularly in patients with chronic diseases, whereas the use of supplementation demands further investigation.

Furthermore, the relation between linoleic (CLA) and linolenic (LNA) acid intake is very important for homeostasis [73]. After being ingested and absorbed, these fatty acids are metabolized and underwent elongation, desaturation, and retroconversion reactions. The action of cyclooxygenase and lipooxygenase enzymes on these fatty acids leads to the formation of different eicosanoids (prostaglandins, leukotrienes, thromboxanes, etc.), whereas the derivatives of the n-3 series are more anti-inflammatory and those of the n-6 series are more proinflammatory. The importance of balance for the n-3 : n-6 ratio is due to the fact that the desaturase *δ*-6 enzyme has greater affinity with LNA than with CLA. Therefore, in order to avoid an imbalance in the production of anti- and proinflammatory eicosanoids, studies suggest that the CLA ratio in the diet is greater than the LNA ratio, whereas the appropriate ratio is around 4 to 5 : 1 and not superior to 10 : 1 [74].

In its turn, the extravirgin olive oil, mainly composed by MUFA (w-9 series)—oleic acid—has reduced the risk of NCD when used as supplement or consumed within the context of healthy dietary patterns [75]. Martínez-González et al. [76] reported a reduction in the risk of atrial fibrillation in individuals with high risk of cardiovascular disease, after intervention with Mediterranean diet supplemented with extravirgin olive oil. In another study, individuals with cardiovascular risk submitted to the Mediterranean diet supplemented with 50 ml/day of extravirgin olive oil reduced the CRP, IL-6, sICAM, and P-selectin concentrations, when compared to the control group, which was provided with a hypolipidemic diet [7]. Ceriello et al. [77] also compared the effect of hypolipidemic and Mediterranean diets, although without oil supplementation, and reported that, after three months, there was a reduction of IL-6, intercellular adhesion molecule 1 (ICAM-1), and 8-isoprostaglandin F2*α* (8-iso-PGF2*α*) only in the group to whom the Mediterranean diet was offered.

Nevertheless, the concentration of phenolic compounds in the oil, capable of reducing the synthesis of AA and cyclooxygenase enzymes 1 and 2 (COX-1 and COX-2), may be a key factor in its potential to reduce inflammatory markers. Camargo et al. [78] compared the effect of three meals prepared with virgin oil classified according to the content of phenolic compounds as high (398 ppm), intermediate (149 ppm), and low (70 ppm). The participants who were provided with meals with high content of phenolic compounds displayed greater reduction in the IL-6, IL-1, and CXC motif chemokine ligand (CXCL) expression than the other groups. Indeed, oil consumption is associated with a lower inflammation degree, particularly in the context of the Mediterranean diet, and its use indicates an important nutritional strategy for the reduction of cardiovascular diseases [79].

The large number of studies devoted to investigate the relation between lipids and inflammation highlights the importance of this macronutrient to the modulation of several inflammatory biomarkers (Table 3). Thus, the literature indicates that adjusting the proportion of SFA, MUFA, and PUFA in the diet is a more effective strategy to modulate the inflammation than the use of hypolipidemic diets, which may play the inverse role. In addition, the higher SFA consumption is related to the increase of inflammatory biomarkers, and this increase is proportional to the ingested amount. Thus, choosing foods rich in MUFA and PUFA and avoiding foods with high SFA content may be beneficial, with respect to the modulation of the inflammation and reduction of the risk of diseases associated with it. Among the alternatives, it is possible to highlight the consumption of extravirgin olive oil with high phenolic compounds content, which supports not only the reduction of inflammation but also the improvement of the endothelial function. Despite the high number of studies available, there is still limited information about the influence of the nutritional state in the modulation of lipids on the inflammation, since obese individuals seem to be less responsive to the benefits of an adequate lipidic distribution.

### 3.3. Proteins, Carbohydrates, and Glycemic Index

Proteins are the macronutrients of greatest thermogenic effect due to their high cost of metabolic synthesis, hydrolysis, gluconeogenesis, and excretion as urea [10]. In addition, the protein content of the diet may lead to increased satiety and decrease in calorie intake [80, 81].

The effects on human health arising from the ingestion of several protein types have been investigated, although their association with metabolic diseases is still conflictive [80]. A study with a Mediterranean population, particularly from Greece, who consumed diets based on plants, vegetables, fruits, and cereals, shortly after World War II, reported lower mortality rates from cardiovascular diseases, due to the low amounts of protein in relation to heme-iron [82]. Nevertheless, in the context of a western diet, a high iron bioavailability was reported, mainly because of the ingestion of heme-iron-related animal protein, which favors an anti-inflammatory state [83].

Currently, iron has been reported as a promoter of atherosclerosis due to its ability to produce free radicals [84]. According to Vallianou et al. [85], in their study with 490 middle-aged Caucasian adults who were apparently healthy—in which they evaluated the protein ingestion of the diet, taking into account the animal and vegetable protein—the serum concentration of cystatin C decreased as the frequency of animal protein increased and the platelet count decreased with the increase of vegetable protein intake, whereas no association was found with monocytes, lymphocytes, and CRP. The influence of protein in the diet on postprandial inflammation is still uncertain. Arya et al. [86] reported that, in healthy individuals, meat with high fat content is more proinflammatory, in comparison with lean meat, in relation to TNF-*α* and IL-6.

Azadbakht and Esmaillzadeh [87] verified that the consumption of red meat was related to higher plasma concentrations of CRP in women. Cocate et al. [88], in a study with 296 men between 40 and 59 years of age, observed a higher occurrence of central obesity, hypertriglyceridemia, and metabolic syndrome in individuals within the first tertile of BCAA (branched-chain amino acids) and leukin consumption, in comparison with those from the second and third tertiles; besides, the higher BCAA and leukin consumption has been considered a protective factor for central obesity and metabolic syndrome. In addition, in another study with the same sample, Cocate et al. [89] verified that the increased consumption of red meat was associated with a higher occurrence of metabolic syndrome and hypertriglyceridemia. Furthermore, a cross-sectional study with 553 individuals aged between 18 and 80 years verified that the consumption of processed meat showed positive association with IL-6, TNF-*α*, TNF-R1, and TNF-R2, even after adjusting for consumption of fruits and vegetables. In turn, the consumption of unprocessed red meat was inversely associated with TNF-R1 and TNF-R2 [90]. Thus, the higher protein and amino acid intake from red meat is directly related to cardiometabolic diseases, whereas the variation of inflammatory markers also seems to be involved.

Dairy products are also a food group deemed as an important protein source. Hence, Zemel et al. [91] found an inverse relationship between the consumption of dairy products and inflammation, both in overweight and obese individuals. In a randomized case-control study, which assessed the relationship between different protein sources, the authors observed that, after intervention, there were lower CC5—chemokine binding to the chemotactic monocyte-1 protein, which facilitates adherence and transmigration of monocytes through the arterial wall, thus leading to an inflammatory process—concentrations in individuals who consumed whey, in comparison with those who consumed cod and casein, in addition to lower CC5 concentrations in individuals who consumed gluten, in comparison to those who consumed cod [92]. In turn, MCP-1 displayed higher values in individuals who consumed whey, in comparison to those who consumed cod and gluten, which can be explained by specific milk properties, such as increased insulin response, as whey is more insulinotropic than cod, gluten, and casein protein. As a general result, whey showed better anti-inflammatory effects, which suggest that this protein source is relevant for the modulation of inflammatory biomarkers.

Amini et al. [93], in a study with 56 Iranian women, engaged in physical activities, found a marginally significant decrease in the CRP concentrations after intervention, both in the hyperproteic (HP) and the balanced (B) diets. This finding, which, unlike the others, did not find any advantage of the HP diet, may be justified by the effect of physical activity, and not only the diets, as a protective factor.

In addition to the studies that assessed the amount and quality of protein associated with the NCD and chronic subclinical inflammation, the quality and amount of carbohydrates or the glycemic index have been highlighted within the scientific literature (Table 4). Cross-sectional studies have demonstrated an inverse association between the intake of whole-grain carbohydrates and low-grade inflammation [94]. However, recent studies about the replacement of refined whole-grain wheat products were inconclusive [95]. Therefore, in general, the consumption of whole-grain products is inversely related to inflammation.

For example, Montonen et al. [96], in a study with 2198 individuals from the European Prospective Investigation into Cancer and Nutrition (EPIC), observed an inverse correlation between the consumption of whole bread and CRP and gamma-glutamyl transferase (GGT) values, as well as a direct correlation between the consumption of red meat and the same inflammatory markers.

With respect to simple carbohydrates, its elevated consumption is worrying, especially when added in foods and drinks, thus favoring the inflammatory state and negative health effects. The World Health Organization (WHO) recommends that children and adults reduce the sugar intake to <10% of total energy, and that this value should be lowered over lifetime [35].

In this context, a study with eutrophic individuals, aged between 20 and 80 years, which aimed to determine the effect of the chronic fructose (55%) consumption, sucrose, and honey on glucose-tolerant (GT), as well as glucose-intolerant (GI) individuals, reported that the GI group displayed serum CRP concentrations > 3 mg/L before and after intervention, and these values were lower to those observed in the acute inflammation, which may indicate an increased risk of cardiometabolic disease [100]. CRP concentrations of the GT group were normal (<3.0 mg/L), although there was a nonsignificant increase after the intervention. IL-6 values were also greater in the GI group. Aeberli et al. [101] observed a CRP elevation after isolated fructose, glucose, or sucrose consumption, while it is important to highlight that the sucrose dose was higher than that of the previous study (80 versus 50 g/d), as was the intervention period (2 versus 3 weeks).

Another study with obese individuals reported higher CRP and lower adiponectin, following hyperglycemic (59–67% of TCV) diets [102]. In turn, hyperglycemic (10–13% of TCV) diets lead to a decrease in the IL-6 concentrations [103]. However, other studies did not report relationships between the amount of carbohydrates and adiponectin [104], IL-6, and CRP concentrations [105].

In turn, with respect to the studies with adults, which associate the ingestion of protein and carbohydrates/glycemic index to the ingestion of hyperproteic (10–15% of TCV), HP (23–28% of TCV) [106], low-glycemic index (LGI), high-glycemic index (HGI) [97], HP + HGI, HP + LGI, LP + HGI, and LP + LGI, in relation to the inflammation, the authors observed that HP diets increased, while B diets decreased, the CRP levels, respectively.

Studies that assessed the effects of GI and protein intake on the inflammatory markers in children are scarce. The study Diet, Obesity, and Genes (Diogenes), found an increase in body fat in children who consumed a HP + HGI diet, whilst the percentage of overweight children decreased in the group who consumed the HP + LGI test diet [107]. In this same study, a decrease was observed in children and adolescents' CRP between 5 and 18 years of age who consumed a LGI diet [106]. This is in contrast with the study by Diogenes with adults, in which CRP concentrations decreased in individuals who consumed hyperproteic diet with HGI [104]. This may be explained by the lower diet adherence by the families and by the fact that the decrease of GI reached less than 4 difference points after the nutritionists' assistance to the families within the study [98].

Thus, it is possible to acknowledge that the lower consumption of protein from red meat and the higher consumption of proteins from vegetables, white meat, and milk displayed beneficial health effects and may lead to the decrease of inflammatory markers. However, there is still lack of consistent information about the effect of protein origin and amount on the inflammation, particularly in healthy people. With respect to GI and carbohydrates, results indicate that the moderate ingestion of carbohydrate, as well as of food with LGI, may have a beneficial effect on the metabolic and inflammatory states in obese individuals, while in children and adolescents, the LGI displayed an inverse effect.

## 4. Conclusions

From this literature review, we reiterated the importance of a varied diet, rich in natural foods and poor in processed foods, such as some previously known patterns (Mediterranean and DASH diets) to prevent and improve low-grade inflammation. With respect to the macronutrients, the provided consumption of PUFAs and MUFAs, as replacements for SFA (moderate intake of carbohydrates, in particular the ones with low glycemic index, and the consumption of proteins, in particular the ones of plant origin and white meats), may positively influence the modulation of low-grade inflammation and, consequently, has a protective effect for the development of the NCD.

However, further studies are necessary, in order to evaluate the inflammatory response to the consumption of dietary patterns or intake of specific macronutrients in different situations of weight, age, and metabolic condition, for a better understanding of the most appropriate dose-response.

## Figures and Tables

**Table 1:** Paper selection stages and inclusion and exclusion criteria for the systematic review.

Stages	Inclusion and exclusion criteria	*N*
1	*Papers identified in the databases*	
	PubMed	4869
	ScienceDirect	4560
	Total number of studies identified	9429

2	*Excluded documents and papers* (*title and abstract screening*)	
	Review papers	1510
	*In vitro* or animal model studies	1277
	Editorials and book chapters	1752
	Studies that did not aim to evaluate the inflammation	3073
	Studies that used registered mark supplements	12
	Studies that assessed pregnancy diet and newborn's inflammation	6
	Studies in which inflammation was related to diseases other than diabetes, dyslipidemia, metabolic syndrome, obesity, and heart diseases	1329
	Studies that did not assess the dietary pattern in general	178
	Duplicated	46
	Total number of papers excluded	9183

3	*Papers selected*	246

4	*Papers excluded* (*full reading*)	
	No results regarding inflammatory markers, only of other cardiovascular risk markers	187
	Results regarding gene expression only	19

5	*Total number of papers included*	40

**Table 2:** Studies that assessed the effect of dietary patterns on the chronic subclinical inflammation.

Reference	Study type	Population	Methods	Results
Ozawa et al. [15]	Cohort	5083 British overweight adult individuals	Validated FFQ; determination of factorial inflammatory loadings for food groups and identified two dietary patterns (pro- or anti-inflammatory)	Dietary pattern considered inflammatory characterized by red meat, fried foods, and lower ingestion of whole grains: ↑ IL-6

Kuczmarski et al. [40]	Cohort	2176 American adult individuals	24 h recalls; identification of the most consumed foods was grouped according to the similarity of their components and new dietary patterns were created; cluster analysis for definition of ten groups	All patterns displayed elevated (>3 mg/L) CRP, whereas the highest average was that of the “frozen food” pattern (7.2 ± 1.4 mg/L)

Corley et al. [13]	Cross-sectional (from a cohort)	792 Scottish elderly eutrophic individuals	Self-applied FFQ; dietary patterns as Mediterranean and conscious (high intake of fruits and carrots and low intake of embedded foods, eggs, pork, and liqueurs)	Highest score of the conscious dietary pattern and higher ingestion of fruits: ↓ CRP; highest score of the Mediterranean dietary pattern: ↓ fibrinogen

Kong et al. [5]	Case-control	45 overweight and obese adults	7-day (for overweight and/obese) and 3-day dietary record (for the control group); three dietary patterns were determined: Group 1 (less healthy, high consumption of beverages and foods high in sugar, fat, and salt); Group 2 (healthier, with higher consumption of water, yogurt, cereals, eggs, and nuts); Group 3 (healthier, with a fiber-rich diet, lower consumption of sugar, fruits, yogurt, and soups)	Group 3: ↓ sDC14, followed by Group 2 and Group 1; Group 3: ↑ DC163; no differences between the groups for the LPS, CRP, and IL-6 concentrations; higher consumption of fruits, green vegetables, and soups ↑ DC163

Lee et al. [32]	Cross-sectional	7574 eutrophic adult individuals	FFQ; 4 dietary patterns determined as standard: fruit, vegetable, meat, and coffee, according to the prevailing food	Highest score for the “vegetable” pattern: ↓ CRP; highest score for the “vegetable” pattern: ↑ ingestion of antioxidants

Bédard et al. [12]	Clinical trial	70 Canadian, overweight, adult individuals, with risk of cardiovascular disease	Four weeks following the Mediterranean pattern	Male individuals who displayed CRP values > 3 mg/L displayed a reduction over time; male individuals who displayed reduced CRP values (<3 mg/L), displayed an increase over time; no beneficial effect of the Mediterranean diet was observed for both genders

Abete et al. [49]	Case-control	51 healthy individuals and 51 individuals with history of stroke	FFQ; 2 dietary patterns determined as “healthy” and “not healthy”	Healthy individuals displayed greater adherence to the healthy dietary pattern, as well as ↓ CRP and ↓ leukocytes

McGeoghegan et al. [44]	Cohort	1531 healthy and diabetic individuals	4-day dietary record during 4 years; 2 dietary patterns determined: “1”—higher antioxidant and anti-inflammatory loads; “2”—lower antioxidant and anti-inflammatory loads	Dietary pattern “1” was inversely related to the CRP concentrations

FFQ: Food Frequency Questionnaire; IL-6: interleukin 6; CRP: C-reactive protein; sDC14: differentiation cluster 14; DC163: differentiation cluster 163; LPS: lipopolysaccharide.

**Table 3:** Studies included in this review, which assessed the effect of lipids on the chronic subclinical inflammation.

Reference	Study type	Population	Methods	Results
Kantor et al. [56]	Retrospective cohort	8177 elderly, institutionalized Americans, included in the 1999–2004 cycles of the National Health and Nutrition Examination Survey	BMI classification: eutrophic < 25 kg/m²; overweight ≥ 25 < 30 kg/m²; obese ≥ 30 kg/m²	Lower SFA intake and utilization of fish oil were associated with ↓ CRP in eutrophic and overweight groups, but not in obese
			24 h recall	

Krysiak et al. [69]	Randomized, controlled clinical trial	101 (66 M/35 W) overweight, hypertriglyceridemic Polish adults	Group 1: placebo	Group 2: ↓ IL-2, IFN-*γ*, and TNF-*α* and CRP n-3 did not significantly reduce these parameters
			Group 2: bezafibrate (200 mg twice a day)	Bezafibrate was better than n-3 in inhibiting low-grade inflammation
			Group 3: n-3 (1 g, twice a day)	
			Duration: 12 weeks	

Dewell et al. [70]	Randomized, controlled clinical trial	100 obese American adults, with metabolic syndrome	Group 1: vegetable-derived n-3	↔ sICAM-1, IL-6, and monocyte chemoattractant protein in all three groups
			Subgroup 1: low dose (2.2 g/d of n-3)	No beneficial independent effect of dose or source
			Subgroup 2: high dose (6.6 g/d of n-3)	
			Group 2: sea-derived n-3	
			Subgroup 1: low dose (1.2 g/d)	
			Subgroup 2: high dose (3.6 g/d)	
			Group 3: placebo	
			Duration: 8 weeks	

Malekshahi Moghadam et al. [61]	Double-blind, controlled, randomized clinical trial	84 (42 M/42 W) overweight Iranian adults and elderly with DM for at least 2 years	Group 1: 3 n-3 capsules/day (EPA 1.548 mg; DHA 828 mg; other n-3 338 mg)	Group 1: ↓ IL-2 and TNF-*α*
			Group 2: 3 placebo capsules (sunflower oil 2100 mg)	↔ CRP
			Duration: 8 weeks	

Kratz et al. [67]	Double-blind, controlled, randomized clinical trial	24 (8 M/16 W) overweight, or level-1 obese American adults	Group 1: n-3-rich diet (3.5% of diet energy)	↔ Gene expression of inflammatory mediators by adipose tissue
			Group 2: diet poor in n-3 (0.5% of energy diet)	↔ IL-6, MCP-1, TNF receptors 1 and 2, and CRP
			Duration: 14 weeks (ad libitum by 12)	

Rajkumar et al. [62]	Randomized, controlled clinical trial	60 (30 M/30 W) overweight Indian adults	Group 1: placebo	Group 2: ↓ CRP
			Group 2: omega-3 (EPA 180 mg/d, DHA 120 mg/d)	Group 4: more pronounced effect in CRP reduction
			Group 3: probiotic VSL # 3 (112.5 × 10^9^ UFC)	
			Group 4: omega-3 (EPA 180 mg/d, DHA 120 mg/d) and probiotic VSL # 3 (112.5 × 10^9^ UFC)	
			Duration: 6 weeks	

Cipollina et al. [66]	Double-blind, controlled, randomized clinical trial	45 overweight American adults, with EPA + DHA consumption ≤ 300 mg/d	Group 1: capsules containing 1.4 g/d of EPA + DHA	Group 1: ↑ DHA and EPA, no significant modulation in the levels of arachidonic acid metabolites
			Group 2: Placebo (soy oil)	
			Duration: 4 months	

Nigam et al. [71]	Double-blind, controlled, randomized clinical trial	316 (221 M/105 W) overweight American adults and elderly with symptomatic paroxysmal or persistent FA	Group 1: fish oil (4 g/d)	Group 1 ↔ inflammation or oxidative stress ↓ CRP in a similar degree between the groups, after 6 months (CRP 11% versus 11% for fish oil versus placebo, respectively)
			Group 2: placebo	
			Duration: 271 ± 129 days	

Ito et al. [63]		125 (64 M/61 W) obese Japanese adults, with (*n* = 94) or without (*n* = 31) dyslipidemia	Group 1 (treatment group—dyslipidemic): diet (isocaloric, normoproteic, and normolipidemic), EPA 1.8 g/d	DHA/AA and DGLA/AA ratios were higher in dyslipidemic obese, in comparison with the nondyslipidemic
			Group 2 (control—dyslipidemic): diet (isocaloric, normoproteic, and normolipidemic), no EPA.	Group 1: ↓ CRP, ↓ DLGA/AA ratio
			Group 3 (control—nondyslipidemic): no intervention	
			Duration: 3 months	

Lee et al. [32]	Double-blind, controlled, randomized clinical trial	14 obese American with metabolic syndrome and 45 adults and elderly type-2 diabetics	Group 1: corn oil (4.02 g/d)	Group 2: ↑ serum ALA
			Group 2: botanical oil (BO) (6.28 g/d)	Group 3: ↑ EPA and DHA; ↓ DGLA and AA
			Group 3: fish oil (FO) (7.64 g/d)	Groups 1 and 2 were associated with increased levels in biomarkers related to type-2 diabetes and metabolic syndrome
			Duration: 8 weeks	

Camargo et al. [78]	Randomized, cross-over clinical trial	49 individuals (19 M/30 W) with metabolic syndrome	Group 1: breakfast rich in olive oil with high phenolic content (398 ppm)	Group 1: higher IL-6, IL-1, CXCL reduction
			Group 2: breakfast rich in olive oil with intermediate phenolic content (149 ppm)	
			Group 3: breakfast rich in olive oil with low phenolic content (70 ppm)	
			Duration: 2-hour postprandial	

Ceriello et al. [77]	Randomized, controlled clinical trial	24 diabetics (17 M/7 W)	Group 1: Mediterranean diet (olive oil)	Group 1: ↓ IL-6, sICAM-1, PGF2*α*
			Group 2: hypolipidemic diet	
			Duration: 3 months	

Casas et al. [7]	Randomized, controlled clinical trial	164 (77 M/87 W) individuals with cardiovascular risk	Group 1: Mediterranean diet + 50 ml extravirgin olive oil	Groups 1 and 2: ↓ CRP, IL-6, sICAM
			Group 2: Mediterranean diet + 30 g/d of oilseeds	
			Group 3: hypolipidemic diet	
			Duration: 12 months	

Darghosian et al. [72]	Double-blind, controlled, randomized clinical trial	190 (109 M/81 W) overweight American adults and elderly with FAR	Group 1: n-3 (4 g/d)	↔ IL-6, IL-8, IL-10, TNF-*α*, monocyte chemoattractant protein, and vascular endothelial growing factor
			Group 2: placebo	
			Duration: 6 months	

Krantz et al. [68]	Double-blind, controlled, randomized clinical trial	72 (22 M/42 W), mainly Latin (71%) obese adults and elderly	Group 1: 3.36 g/d of n-3 (EPA/DHA)	Group 1: nonsignificant CRP reduction
			Group 2: placebo	↔ Inflammation
			Duration: 3 months	

Itariu et al. [64]	Randomized, controlled clinical trial	55 (9 M/46 W) level-3 obese American adults and elderly	Group 1: 3.36 g/d of n-3 (EPA/DHA)	Group 1: ↓ gene expression of inflammatory markers in subcutaneous adipose tissue
			Group 2 (placebo): 3.36 g/d of butter	↓ IL-6
			Duration: 8 weeks	↑ anti-inflammatory eicosanoids in visceral adipose tissue

Lesná et al. [57]	Double-blind, controlled, randomized clinical trial	15 overweight or obese adult American women, in postmenopause	2- to 3-week cross-over interventions, with 1-week interval between them	Change from Group 1 to Group 2: ↓ CRP and IL-18 (the latter was not significant)
			Group 1 (animal-derived SFA): 42% of TCV	
			Group 2 (vegetable-derived PUFA): 40% of TCV	

Silver et al. [59]	Double-blind, controlled, randomized clinical trial	144 level-1 obese adult American women in postmenopause	Women received Group 1 diets during 2 weeks	↓ IL-1*α*, IL-1*β*, 1L-12, IL-17, IFN-*γ*, TNF-*α*, TNF-*β*
			Group 1 (DHL): high-fat diet (50% of TCV); 1/3 SFA, 1/3 MUFA, 1/3 PUFA)	Compared to DHL + P, DHL + S had higher effect on the IFN-*γ* reduction (↓74%) and DHL + L had higher effect on PAI-1 reduction (↓ 31%)
			Hence, they were randomized into the following groups for 14 weeks:	MUFA/PUFA/SFA in the diet changed markers to CVD
			Group 2 (DHL-P): DHL + placebo	
			Group 3 (DHL-S): DHL + stearate (9 g/d)	
			Group 4 (DHL+O): DHL + oleate (9 g/d)	
			Group 5 (DHL+L): DHL + linoleate (9 g/d)	

Agh et al. [65]	Double blind, controlled, randomized clinical trial	45 men with coronary artery disease	Group 1: n-3 (720 mg EPA, 480 mg DHA)	Group 1: ↓ CRP
			Group 2: placebo (edible paraffin)	
			Duration: 8 weeks	Group 2: ↔ CRP

M: men; W: women; BMI: body mass index; SFA: saturated fatty acids; CRP: C-reactive protein; n-3: omega-3; sICAM-1: soluble intercellular adhesion molecule-1; IL: interleukin; IFN-*γ*: interferon gamma; TNF-*α*: tumor necrosis factor alpha; EPA: eicosapentaenoic acid; DHA: docosahexaenoic acid; DM: diabetes mellitus; AA: arachidonic acid; DGLA: dihomo-gamma-linolenic acid; ALA: alpha-linolenic acid; CXCL: chemokine (C-X-C motif) ligand; PGF2*α*: prostaglandin F2*α*; PAI-1: plasminogen activator inhibitor-1; CVD: cardiovascular disease; MUFA: monounsaturated fatty acids; PUFA: polyunsaturated fatty acids.

**Table 4:** Studies addressing the effect of proteins, carbohydrates, and glycemic index on the chronic subclinical inflammation.

Reference	Study type	Population	Methods	Results
Azadbakht et al. [88]	Cross-sectional	482 female teachers from Tehrani, between 40 and 60 years old	(i) Usual dietary intake was assessed through a semiquantitative FFQ with 168 items	PCR plasma concentrations were higher in individuals from higher quintiles of red meat consumption, even after model fit
			(ii) FFQ foods were classified into 41 food groups, based on the nutrient profile, culinary, or specific use	
			(iii) Red meat category was defined by the sum of processed meat (sausages and hamburgers), red meat (beef and lamb), and organ meat (liver, kidney, and heart)	
			(iv) Blood samples: assess inflammatory markers	

Vallianou et al. [85]	Cross-sectional	490 middle-aged Caucasian adults, apparently healthy	(i) Validated semiquantitative FFQ, with 76 food items	*Unadjusted model*:
		BMI: between 26 and 27 kg/m²	(ii) Food items were grouped according to the protein content: nonheme (dairy products and eggs), heme (meat and by-products, fish and seafood), and vegetable protein	*Cistatin C*: decrease in serum levels as frequency of protein animal heme consumption increased
			(iii) Blood samples taken after 12 hours of fasting: cystatin C, CRP, white cells, uric acid, and platelets as inflammatory parameters	*Platelets*: reduction of counting with the increase of vegetable protein consumption
				*Model adjusted to*: vegetable protein intake + energy intake + age + gender + BMI + smoking habit + physical activity
				*Platelets*: reduction of counting with the increased frequency of vegetable protein consumption

Montonen et al. [96]	Cross-sectional	2198 German individuals from the EPIC cohort (836 M, between 40 and 65 years old; 1362 W, between 35 and 65 years old)	(i) Usual intake of previous year was assessed through a self-applied FFQ, with 148 items	(i) Inverse association of whole bread consumption with [CRP] and [GGT]
		BMI: between 25 and 26 kg/m²	(ii) Frequency of consumption was requested in 10 categories, and the amount of consumption was calculated in grams	(ii) Direct association of red meat consumption with [CRP] and [GGT]
			(iii) Reproducibility after 6 months: high for red meat intake and moderate for whole bread intake	
			(iv) Intake of these foods was divided in quintiles	
			(v) Blood samples: assess CRP was inflammatory parameter and GGT for oxidative stress	

Cocate et al. [87]	Cross-sectional	296 men, working at the Universidade Federal de Viçosa (Brazil), between 40 and 59 years old	(i) Usual dietary intake was assessed through an FFQ, validated for the Brazilian population	(i) Ox-LDL concentrations displayed positive correlation with red meat consumption and the saturated fat in it
			(ii) The red meat group was composed of lean meat, high-fat meat, ground beef, lean pork meat, high-fat pork meat, and bacon	
			(iii) The white meat group was composed of chicken with skin, skinless chicken, and fish	
			(iv) Blood samples after 12 h of fasting: assess ox-LDL	

Schwedhelm et al. [90]	Cross-sectional	553 individuals from the Bavarian Food Consumption Survey II group, between 18 and 80 years old	(i) Dietary intake, including meat intake, was assessed through three 24 h recalls (2 in weekdays and 1 in the weekend)	(i) Processed meat consumption displayed positive association with IL-6 after adjusting for fruit and green vegetable consumption, except when there was BMI addition to the model
			(ii) Red meat: beef, veal, pork, mutton or lamb, and domestic and game rabbit	(ii) Processed meat consumption was positively associated with TNF-*α*, TNF-R1, and TNF-R2, even after adjusting for fruit, green vegetable, and dairy consumption
			(iii) Processed meat: ready-made meat or meat preserved by salting, smoking, curing, marination, or cooking	(iii) Consumption of nonprocessed red meat was inversely associated with TNF-R1 and TNF-R2
			(iv) Blood samples: assess CRP, IL-6, total TNF-*α*, and TNF-R1 as inflammatory parameter	

Arya et al. [86]	Randomized cross-over	10 (6 M/4 W) healthy individuals between 19 and 38 years old	Before testing diets: 10 h fasting	Fat meat is more proinflammatory than lean meat:
		BMI: between 18 and 26.5 kg/m²	Types of meals:	(i) 1 h postprandial: [CRP], [TNF-*α*], and [IL-6] were significantly greater in the group who consumed fat meat
			(i) Lean meat (<4% fat, whereas <1% saturated fat) + 75 g of boiled potato + 50 g of peas	(ii) 2 h postprandial: [TNF-*α*] and [IL-6] were significantly greater in the group who consumed fat meat
			(ii) Fat meat (25–30% fat, whereas 40% saturated fat + 75 g of boiled potato + 50 g of peas	
			Participants were categorized in 2 groups of 5, and each group received a type of meal. After 6–10 days, group meals were changed	
			Blood samples 1 h and 2 h postprandial: assessment of CRP and TNF-*α* as inflammatory parameters	

Zemel et al. [91]	Randomized cross-over	20 healthy adults (14 M/6 W), professors, employees, and students at the University of Tennessee, with mean age = 31 years	(i) The study displayed 2 weight-maintenance diets: one for the overweight group and the other for the obese group	Skimmed milk smoothie significantly decreased circulating TNF-α (15%) and IL-6 (13%), whereas soybean diet had the opposite effect
		BMI: 10 overweight (25.0–29.9 kg/m²) and 10 degree-1 obesity (30–34.9 kg/m²)	(ii) Both diets offered ≈35% fat, ≈49% carbohydrates, 16% protein, and 8–12 g/d of fiber; the placebo diet was based on soy protein and the testing diet on skimmed milk protein	MCP-1 concentrations were significantly lower in individuals who consumed skimmed milk smoothie, whereas the soy smoothie caused an increase in MCP-1
			(iii) Both groups were categorized in two additional ones and each subgroup received for 28 days: 3 soy protein smoothies or 3 skimmed milk protein smoothies. After 28 days, subgroups meals were changed for additional 28 days	Skimmed milk smoothie resulted in a significant increase in circulating adiponectin (20%), whereas soy smoothie resulted in its decrease
			Each smoothie shake contained 170 kcal, 10 g of proteins, 1 g of fat, and 30 g of carbohydrates	CRP exhibited an global treatment effect, which resulted in a significant reduction (57%) through the ingestion of skimmed milk smoothie
			(iv) Blood samples: assess inflammatory markers	

Holmer-Jensen et al. [92]	Randomized case-control	11 Caucasian middle-aged obese individuals (3 M/8 W), BMI: ≥30 kg/m²	*Before testing diets:*	^∗^ *CCL5*
			Standard dietary intake:	CCL5 was higher in the postprandial period of 30 min than in the basal, for all protein sources
			(i) 56% carbohydrates, 24% fat, and 20% proteins	After 4 h postprandial:
			(ii) 1673 kcal women and 2151 kcal men blood sample after 12 h fasting testing diet intake for 20 min	Lower CCL5 for whey protein than to cod and casein protein
			(iii) Hypercaloric: 19% carbohydrates, 66% fat, and 15% protein: 1188–1191 kcal	Lower CCL5 for gluten protein than to cod protein
			(iv) 45 g white bread, 100 g butter, 45 g of protein (mixed with the meal or the water, according to the source) blood samples after 4 h postprandial: analyze inflammatory markers	^∗^ *MCP-1*
				MCP-1 was lower in the postprandial period of 30 min than in the basal, for all protein sources
				After 4 h postprandial:
				Higher MCP-1 for whey protein than for cod and gluten protein

Gögebakan et al. [97]	Randomized case-control	773 European obese adults, with mean age of 41 years	After 8 weeks under low-caloric diet (800 kcal/day), were selected those whose weight loss is ≥8%, and the LC diet was applied again	(i) After low-caloric diet, there was a decrease in [CRP]
		BMI: 34 kg/m², from the Diogenes project	Diet composition:	(ii) During the dietary intervention, there was a decrease in [CRP]: > in the LGI than in the HGI group and > in the LP than in the HP group
			(i) LP (10–15% energy) + LGI	(iii) LGI group displayed an additional reduction of 15% in [CRP]
			(ii) LP + HGI	(iv) After the 26 weeks, the LGI and LP groups displayed significant reduction in [CRP] with respect to the HGI and HP groups
			(iii) HP (23–28% energy) + LGI	
			(iv) HP + HGI	
			(v) Control	
			(a) There should be ≠ 15 points from HGI to LGI	
			(b) Guidance for healthy choices	
			Blood samples after 10 h fasting: CRP assessment as inflammatory parameter	

Damsgaard et al. [98]	Randomized case-control	253 European children and adolescents, from 5 to 18 years old, with overweight parents, from the Diogenes project	Diet composition:	Changes in [CRP] after intervention were greater in the LGI than in HGI, but significance was lost after Bonferroni correction, which may explained by the families with lower adherence to the diets
		4.8% W and 4.2% M, from 10 to 18 years old → MS	(i) LP (10–15% energy) + LGI	
			(ii) LP + HGI	
			(iii) HP (23–28% energy) + LGI	
			(iv) HP + HGI	
			(a) There should be ≠ 15 points from HGI to LGI	
			(b) Guidance for healthy choices	
			Blood samples after 4 h fasting: CRP assessment as inflammatory parameter	

Raatz et al. [99]	Randomized cross-over	55 American eutrophic and obese individuals (16 M/39 W), from 20 to 80 years old	(i) Individuals received the 50 g of three types of sugar to be consumed diluted in water	Basal levels:
		BMI: 18 to 39.9 kg/m²	(ii) Each type of sugar was consumed during 2 weeks, with a 2- to 4-week period between treatments	(i) [PCR] and [IL-6] were significantly greater in TGI
			(iii) Blood samples after 10 h fasting: days 0 and 15 from each experimental period, to assess CRP and IL-6 as inflammatory parameters	Posttreatment:
				(i) [PCR] and [IL-6] were significantly greater in TGI

Amini et al. [93]	Randomized case-control	56 Iranian women, between 20 and 46 years old, BMI ≥ 25 kg/m², engaged in physical activities three times a week, 60 minutes per session	Female volunteers were grouped equally and randomly in two diet groups:	There was a marginally significant decrease in CRP levels after intervention, both in the HP and LP diets
			(i) HP diet: 45% carbohydrates, 25% protein, and 30% fat	
			(ii) LP diet: 55% carbohydrates, 15% protein, and 30% fat	
			(iii) All of them displayed a reduction of 500 kcal from the conventional diet	
			Dietary intake was determined by a food record applied on baseline and every 2 weeks, referring to 3 days of the week	
			(iv) HP and LP diets were provided in an 8-week period	
			(v) Blood samples: assess CRP as inflammatory parameter	

BMI: body mass index; kg/m²: kilogram per square meter; FFQ: Food Frequency Questionnaire; CRP: C-reactive protein; M: men; W: women; CCL5: CC chemokine ligand-5; MCP-1: monocyte chemoattractant protein-1; MS: metabolic syndrome; GI: glycemic index; CVD: cardiovascular disease; LP: low protein; LGI: low glycemic index; HGI: high glycemic index; HP: high protein; kcal: kilocalorie; GGT: gamma glutamyl transferase; IL-6: interleukin 6; GI: glucose intolerance; ox-LDL: oxidized low-density lipoprotein; TNF-*α*: tumor necrosis factor-*α*; TNF-α-R1: tumor necrosis factor receptor 1; TNF-α-R2: tumor necrosis factor receptor 2; B: balanced.
