# Corrigendum to “Dietary Pattern and Macronutrients Profile on the Variation of Inflammatory Biomarkers: Scientific Update”

**DOI:** 10.1155/2018/9830287

**Published:** 2018-12-16

**Authors:** Brenda Kelly Souza Silveira, Thatianne Moreira Silva Oliveira, Patrícia Amaro Andrade, Helen Hermana Miranda Hermsdorff, Carla de Oliveira Barbosa Rosa, Sylvia do Carmo Castro Franceschini

**Affiliations:** Department of Nutrition and Health, Universidade Federal de Viçosa, Viçosa, MG, Brazil

In the article titled “Dietary Pattern and Macronutrients Profile on the Variation of Inflammatory Biomarkers: Scientific Update” [1], a study by Silver et al. [2] was cited in Section 3.2 and it was stated that subjects following a balanced high-fat diet saw “an average reduction of 2.5 kg of body fat, an average increase of 2.5 kg of lean mass.” This was not accurate as Silver et al. reported a decrease in Total Lean Mass over the course of their intervention and changes in relative body composition (%). The real change was a decrease in fat mass (−2.5 ± 2.1 %) to increase in relative lean body mass (+2.5 ± 2.1 %).
